# Automatic Identification of Lower-Limb Neuromuscular Activation Patterns During Gait Using a Textile Wearable Multisensor System

**DOI:** 10.3390/s26030997

**Published:** 2026-02-03

**Authors:** Federica Amitrano, Armando Coccia, Federico Colelli Riano, Gaetano Pagano, Arcangelo Biancardi, Ernesto Losavio, Giovanni D’Addio

**Affiliations:** 1 Movement Analysis and Robotics Laboratory (MARLab), Istituti Clinici Scientifici Maugeri IRCCS, Telese Terme Institute, 82037 Benevento, Italy; federica.amitrano@icsmaugeri.it (F.A.); federico.colelliriano@icsmaugeri.it (F.C.R.); arcangelo.biancardi@icsmaugeri.it (A.B.); gianni.daddio@icsmaugeri.it (G.D.); 2 Movement Analysis and Robotics Laboratory (MARLab), Istituti Clinici Scientifici Maugeri IRCCS, Bari Institute, 70124 Bari, Italy; gaetano.pagano@icsmaugeri.it; 3 Neurology Unit, Istituti Clinici Scientifici Maugeri IRCCS, Bari Institute, 70124 Bari, Italy; ernesto.losavio@icsmaugeri.it

**Keywords:** wearable sensors, e-textile, textile-based electrode, surface electromyography (sEMG), gait analysis, plantar pressure sensors, muscle activation patterns, wearable health monitoring, neuromotor rehabilitation

## Abstract

Wearable sensing technologies are increasingly used to assess neuromuscular function during daily-life activities. This study presents and evaluates a multisensor wearable system integrating a textile-based surface Electromyography (sEMG) sleeve and a pressure-sensing insole for monitoring Tibialis Anterior (TA) and Gastrocnemius Lateralis (GL) activation during gait. Eleven healthy adults performed overground walking trials while synchronised sEMG and plantar pressure signals were collected and processed using a dedicated algorithm for detecting activation intervals across gait cycles. All participants completed the walking protocol without discomfort, and the system provided stable recordings suitable for further analysis. The detected activation patterns showed one to four bursts per gait cycle, with consistent TA activity in terminal swing and GL activity in mid- to terminal stance. Additional short bursts were observed in early stance, pre-swing, and mid-stance depending on the pattern. The area under the sEMG envelope and the temporal features of each burst exhibited both inter- and intra-subject variability, consistent with known physiological modulation of gait-related muscle activity. The results demonstrate the feasibility of the proposed multisensor system for characterising muscle activation during walking. Its comfort, signal quality, and ease of integration encourage further applications in clinical gait assessment and remote monitoring. Future work will focus on system optimisation, simplified donning procedures, and validation in larger cohorts and populations with gait impairments.

## 1. Introduction

Wearable technologies constitute a pivotal and rapidly evolving domain within biomedical engineering, offering transformative potential in the acquisition and analysis of physiological signals. These devices enable the continuous monitoring of biosignals and the extraction of clinically relevant parameters, often in real time, thereby facilitating timely and informed decision-making in both clinical and research contexts. A typical wearable biomedical device comprises two fundamental components: a sensorised module and a textile-based substrate. The integration of conductive fabrics into the textile structure has led to the emergence of electronic textiles (e-textiles), which combine sensing capabilities with enhanced comfort and wearability. This dual functionality renders wearable systems particularly advantageous when compared to conventional instrumentation, which is often cumbersome and requires extensive setup procedures. The ease of use and portability of wearable devices significantly broaden their applicability, especially in domains such as gait analysis and neuromuscular assessment [1]. Gait analysis plays a crucial role in clinical and research settings, as it provides objective measures of locomotor function and is widely used to evaluate rehabilitation outcomes. In particular, the analysis of muscle synergies during gait is essential for understanding motor control strategies, especially in neurological disorders such as stroke, cerebral palsy, and Parkinson’s disease [2,3,4]. In a broader sense, sEMG facilitates the detection of muscular imbalances and aberrant motor patterns, contributing valuable insights into the biomechanics of movement [5]. These insights are instrumental in monitoring muscle fatigue and assessing the efficacy of rehabilitative protocols, making them key outcome measures in neurorehabilitation [6]. The variability of muscle activation patterns observed in healthy gait, as discussed later in this manuscript, underscores the complexity of neuromuscular control and the need for personalised analysis. Electromyography (EMG) signals may be acquired via intramuscular electrodes or through surface electrodes; although the former offer superior signal fidelity, surface electrodes provide a non-invasive alternative that is suitable for wearable applications, with only a modest compromise in precision [7,8]. sEMG has demonstrated extensive utility across various clinical and rehabilitative domains [9]. In the present study, we employed an e-textile sleeve to record the activity of the TA and GL muscles during gait in healthy individuals. The design and validation of the device were presented in a previous work [10]. Participants were equipped with two wearable sensing devices: an e-textile leg sleeve and a sensorised insole, both connected to the same central electronic unit. The sleeve captured sEMG in real time via surface electrodes, while the insole incorporated pressure sensors to correlate muscular activity with gait phases. A dedicated data processing pipeline was developed to extract relevant parameters from the acquired signals, enabling a comprehensive analysis of muscle activation throughout the gait cycle. Although all lower-limb muscles contribute to gait, this study focuses on the TA and GL muscles because of their central role in ankle dorsiflexion and plantarflexion [11]. Dysfunction of dorsiflexor muscles often leads to foot drop, a common complication in neuromotor disorders that significantly impairs gait and increases fall risk [12]. This aspect is a major target in rehabilitation programs, and its monitoring is clinically relevant for guiding treatment strategies. In a broader sense, the purpose of this work is to demonstrate a minimally invasive research option that enables the analysis of selected muscle groups of interest. Extending this approach to other muscles is primarily a matter of sensor placement and configuration, which makes the proposed system adaptable to different clinical and research needs. This integrated approach aims to provide a foundation for future clinical applications, where wearable systems can support both in-lab and remote monitoring of gait-related neuromuscular function.

## 2. Materials and Methods

**Figure 1:**
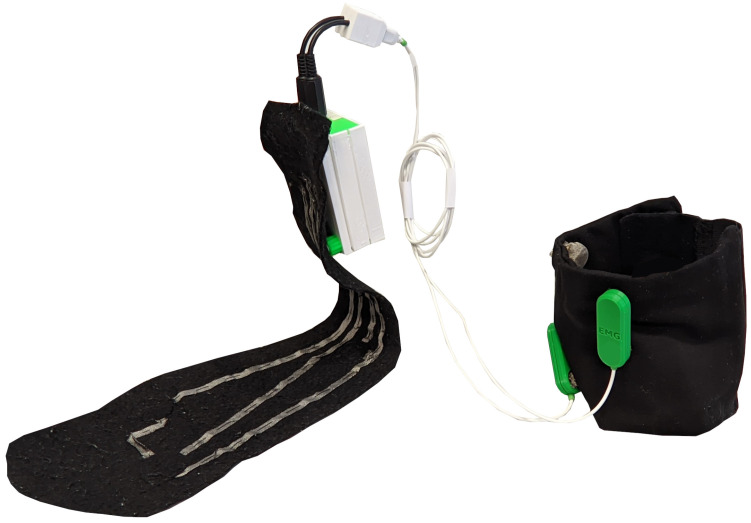
Complete wearable multisensor system configuration that includes the textile leg sleeve with embedded fabric electrodes for sEMG acquisition, the textile insole with integrated pressure sensors, and the central electronic unit connecting both sensing modules.

In this work, a novel wearable system is used to acquire sEMG and foot pressure signals during gait. The system is made up of (i) a textile leg sleeve with embedded fabric electrodes; (ii) sEMG probes; (iii) a textile wearable insole with embedded fabric pressure sensors; (iv) a central electronic device and (v) a mobile application for the management of the acquisition session and for the storage of acquired data. In Figure 1 the wearable system is shown.

### 2.1. Wearable Textile Band for Surface Electromyography

**Figure 2:**
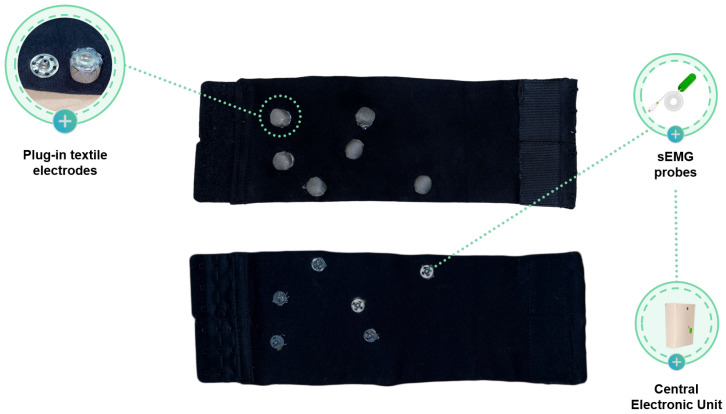
e-Textile sEMG band used for muscle signal acquisition. The figure shows the adjustable elastic sleeve with integrated removable textile electrodes, including four active and two reference electrodes mounted on soft pads.

sEMG signals were acquired using a wearable textile band specifically designed to ensure comfort, adaptability, and reusability (Figure 2). The device consists of an adjustable elastic sleeve incorporating removable textile electrodes, allowing adaptation to different calf circumferences and enabling washing after electrode removal.

The electrodes, made of silver fiber knitted conductive fabric, are mounted on soft non-conductive pads to improve skin adhesion and reduce contact impedance. Their placement follows Surface Electromyography for the Non-Invasive Assessment of Muscles (SENIAM) guidelines for the TA and GL muscles. The system includes four active electrodes (10 mm diameter) and two reference electrodes (15 mm diameter), connected through metal clips compatible with standard sEMG acquisition systems. The external clips allow connection to the acquisition unit via conventional electrode cables, maintaining compatibility with established protocols.

The choice of this solution over traditional pre-gelled electrodes was motivated by several factors: improved user comfort, elimination of single-use consumables, and suitability for long-term or out-of-lab monitoring scenarios. These aspects are particularly relevant for clinical and rehabilitation applications where ease of use and patient compliance are critical.

In addition to technical validation, the device wearability and comfort were assessed using the Comfort Rating Scale (CRS), confirming high user acceptance and absence of discomfort during use. For further details on design, materials, and validation procedures, refer to our previous work [10].

Two EMG Sensor modules (assembled version) from BITalino (PLUX Wireless Biosignals S.p.A., Lisbon, Portugal) were connected to the textile band for signal recording. These sensors are designed for high-quality sEMG acquisition and were interfaced with the central electronic unit for signal acquisition.

### 2.2. Pressure Sensors Wearable Insole

As part of the same wearable system for gait assessment, a textile insole integrating three pressure sensors was employed. This integration is crucial to correlate the muscle activation with the steps during gait as reported in previous works [13,14]. The insole incorporates three circular pressure sensors (diameter 1 cm) positioned at key points of the plantar arch where body weight is distributed: the heel, the first, and the fifth metatarsal bones. These sensors are made of EeonTex™ fabric (Eeonyx Corp., Pinole, CA 94564, USA), a conductive and nonwoven microfiber with piezo-resistive functionality (surface resistivity 2000 ohm/sq), they offer a reduction of the electrical resistance to the application of force. They were embedded in a felt insole that can be worn inside the shoe and easily removed and washed, ensuring reusability and hygiene.

The textile pressure sensors are connected to the conditioning circuit via 5 mm wide conductive ribbon (Adafruit Inc., New York, NY, USA), with a resistance of less than 0.1 ohm per cm. Four connections are provided: one common line for all sensors and three individual lines corresponding to the three sensing points. Each tape end is equipped with a sewn clip to allow connection to the conditioning circuit.

The analog conditioning circuit converts the resistance variation of the textile sensors under pressure into a measurable voltage signal suitable for acquisition by a microcontroller. Specifically, the circuit includes three resistive dividers, each associated with one sensor, enabling simultaneous monitoring of plantar pressure at the selected points.

**Figure 3:**
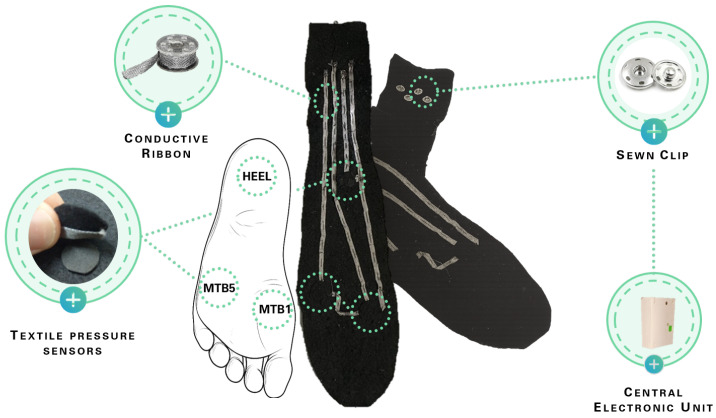
Textile insole integrating three pressure sensors and conductive connections. The central part shows the felt insole with the three EeonTex-based circular sensors positioned at the heel, first metatarsal, and fifth metatarsal. The enlarged views highlight the 5 mm conductive ribbon used for signal transmission, the sewn textile interfaces, and the snap connectors enabling attachment to the conditioning circuit.

The structure of the textile insole, including the placement of the three EeonTex™ pressure sensors and the conductive-wire connections, is illustrated in Figure 3.

### 2.3. Central Electronic Unit

The wearable system integrates a compact central electronic unit designed to acquire analog and digital signals from the sensors and transmit them wirelessly to a mobile application for real-time monitoring and data storage. Communication is based on Bluetooth Low Energy (BLE), ensuring low power consumption and stable connectivity.

**Figure 4:**
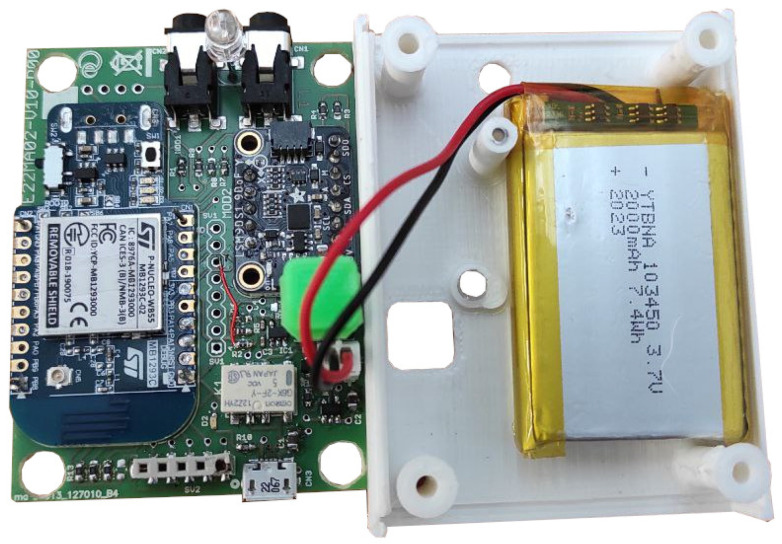
Internal view of the central electronic unit, showing the STM32WB-based acquisition board (**left**) and the integrated LiPo battery within its housing (**right**).

The unit is built around an STM32WB microcontroller (UFQFPN48 package, STMicroelectronics, Geneva, Switzerland), which combines signal processing and wireless transmission in a single component. The electronic unit also includes a Lithium Polymer (LiPo) battery, pressure signals conditioning circuits, an Inertial Measurement Unit (IMU), two jack sockets as interfaces with sEMG probes, four male clips for the connection with the wearable insole, power button and LEDs. This design approach ensures that the entire system remains lightweight and unobtrusive, making it suitable for wearable applications during gait analysis. Signals from the IMU were not employed in the current study; however, the sensor was integrated into the electronic unit to enable its possible use in more in-depth gait analysis. In future applications, kinematic signals could be exploited to enhance the characterisation of gait, by including also spatio-temporal parameters. An overview of the central electronic unit is shown in Figure 4.

### 2.4. Experimental Setup

The study involved eleven healthy subjects (4 women and 7 men; age: 28.09±3.36 years; height: 174±7.8 cm; weight: 69.5±12.7 kg; BMI: 22.8±3.6). The study was conducted in accordance with the Declaration of Helsinki with the approval of Local Ethics Committee (protocol N. 313/2024, study N. 1610/CEL), and informed consent was obtained from all subjects involved in the study. All acquisitions were performed on the right leg and right foot to ensure consistency across participants. Each trial began with a short preliminary phase designed to establish a baseline sEMG. Using the application’s timer, the subject remained upright and still for five seconds before receiving the start signal to begin walking on a straight path of ten meters. This static period provided an sEMG signal close to resting conditions, used to correct the offset during data processing. Although the upright posture requires minimal muscular effort, this was considered negligible compared to the activity recorded during gait. Each subject performed five repetitions of the walking task.

During the trials, the textile sEMG sleeve was worn below the knee on the examined leg, while the pressure-sensing insole was inserted into the shoe. Both the sensing units were managed by the central unit, ensuring synchronization between pressure and EMG data for further processing. The central electronic unit was secured to the leg using an elastic sleeve. Data collected by the unit were transmitted via BLE to a smartphone running a dedicated application, which recorded each walking session, digitised the signals into structured packets and stored them as comma separated values (CSV) files in the internal memory of the device. At the end of each acquisition, the files were transferred to a computer for subsequent analysis.

**Figure 5:**
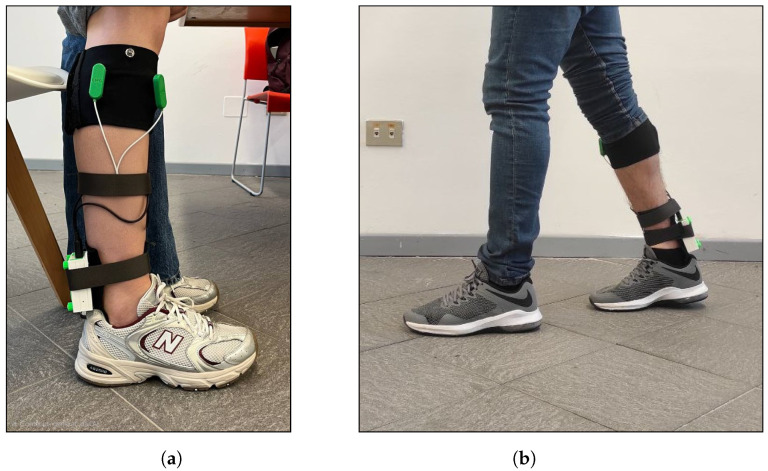
Experimental set-up for acquisition of TA and GL sEMG and plantar pressure signals. Panel (**a**) shows the positioning of the textile sEMG sleeve, the central electronic unit and the sensor connections on the right leg. Panel (**b**) illustrates a representative frame of the walking task with the complete wearable system in place.

Figure 5 illustrates the sensor connections to the acquisition system and the correct positioning of the instrumentation on the subject prior to task execution.

### 2.5. Data Processing

Data analysis was performed using a custom algorithm developed in Python (v3.11) to automatically identify muscle activations and gait events during the walking task.

**Figure 6:**
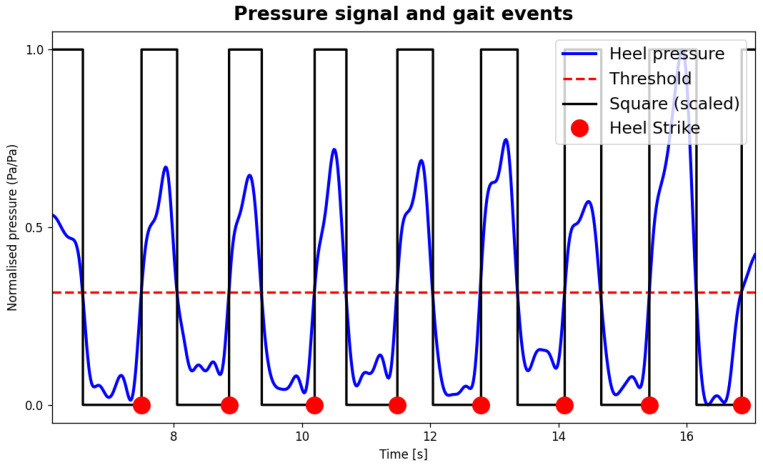
Representation of pressure signal processing. The red-dashed threshold was applied on the filtered heel pressure signal to produce a foot-switch square wave. The instant at which heel pressure overcomes the threshold corresponds to the heel strike event.

Pressure signals were filtered (Butterworth, 5th order, 4 Hz cutoff frequency) and a threshold was applied to detect heel strikes and segment individual steps. Amplitude threshold value was manually set for each trial by an operator through a user interface displaying the recorded pressure data. Figure 6 shows the filtered pressure signal from heel sensor, the threshold applied, and the gait events identified to segment steps.

**Figure 7:**
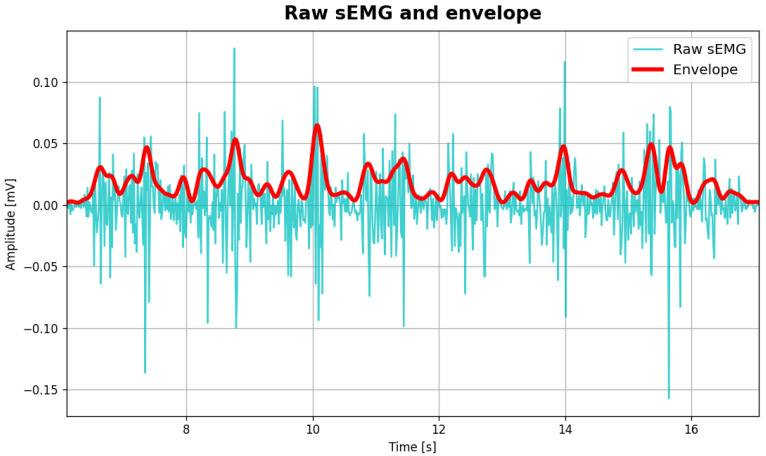
Example of raw sEMG signal acquired from TA muscle in a walking trial and linear envelope (red).

**Figure 8:**
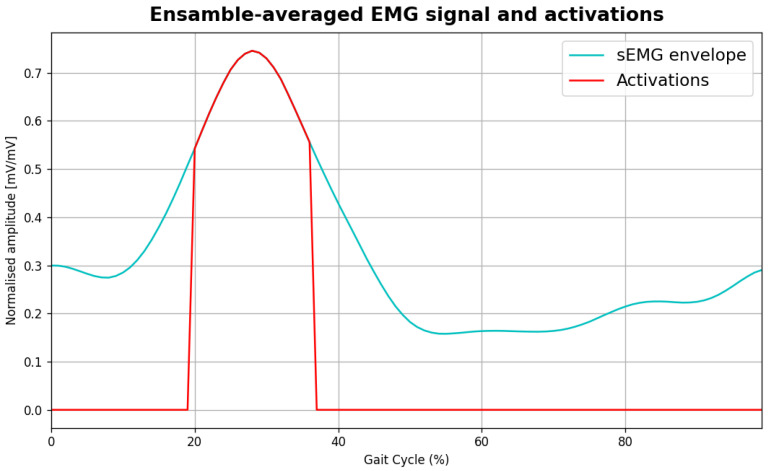
Example of ensamble-averaged EMG signal from GL and detected activations superimposed in red.

The processing workflow of EMG included signal rectification and low-pass filtering (Butterworth, 4th order, 4 Hz cutoff frequency) to compute linear envelopes, as shown in Figure 7. The signal was windowed in successive steps using heel strike events detected in pressure signals, followed by amplitude [15] and time normalisation to ensure comparability across steps. For each walking task, normalised envelopes were averaged to obtain an ensemble-averaged EMG, representative of the TA and GL muscles. Muscle activation was detected using a statistical threshold-based criterion, as commonly adopted in EMG onset detection methods [16]. Specifically, the muscle was considered active when the envelope of the sEMG signal exceeded three times the standard deviation of the signal for at least 80 ms. This approach overcomes typical challenges related to high resting muscle activity or pathological motor activities like tremor, leading to increased false alarms (type I errors), missed detections (type II errors) [17] and errors arising in simpler methods, such as the single-threshold method [18]. Figure 8 shows an example of ensamble averaged EMG signal from GL with the detected activations superimposed in red.

Quantitative parameters were extracted from the ensemble-averaged sEMG signals: (i) number of activation identified in the gait cycle; (ii) onset and offset time of the activations (in percentage of the gait cycle time (GCT)); (iii) total duration of the activations (in percentage of the GCT) and (iv) area under curve during activation time (in percentage of the area under the entire averaged sEMG signal in a gait cycle).

## 3. Results

All participants successfully completed all walking trials without reporting any difficulty or interruption. Throughout the experimental sessions, the wearable system, including the e-textile sEMG sleeve and the pressure-sensing insole, was perceived as comfortable and non-invasive. No subject reported discomfort, skin irritation or limitations in natural gait and none experienced issues related to stability or device-induced constraints during locomotion.

Overall, the analysis highlighted substantial inter- and intra-subject variability in both muscles, with activation intervals ranging from one to four per gait cycle. Despite this variability, recurrent activation patterns, characterised by different numbers and distribution of activations, could be identified for both the TA and the GL.

**Figure 9:**
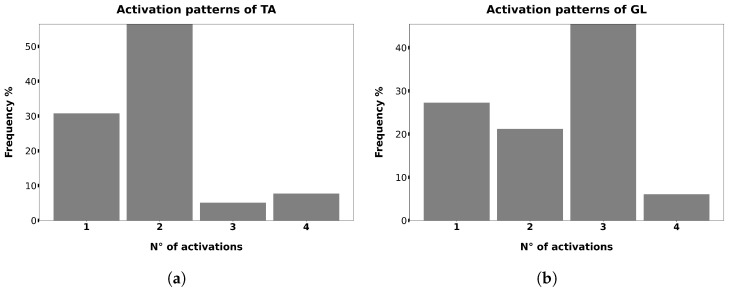
Frequency distribution of the activation patterns observed during the gait cycle. Panel (**a**) reports the distribution for the TA muscle, whereas panel (**b**) reports the corresponding distribution for the GL muscle.

Figure 9 reports the frequency distribution of activation patterns for the two muscles. For the TA, the most prevalent activation mode consisted of two distinct activations per gait cycle (56% of trials), while for the GL the most frequent pattern corresponded to three activations per cycle (46% of trials). These modes represent the activation patterns observed in the highest number of trials across all subjects. The second most recurrent pattern registered for the TA muscle encompasses one activation (31% of trials) while 3- and 4-activations patterns are observed in 5% and 8% of trials respectively. In the analysis of data from the GL muscle we found one activation in 27% of gait trials, two activations in 21% of trials, while 4-activations pattern was observed in only 6% of cases.

**Figure 10:**
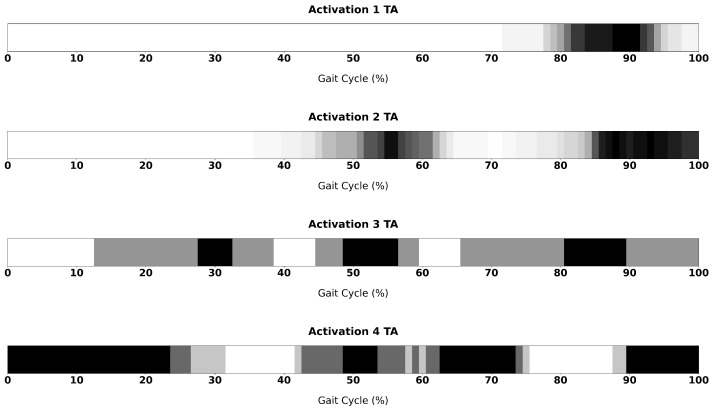
Temporal distribution of TA activation across the gait cycle for subjects exhibiting one to four activation intervals. Each row represents an activation modality, while the grayscale maps indicate the density of ON periods across the normalised gait cycle (%GCT). Darker regions correspond to higher activation frequency among subjects.

**Figure 11:**
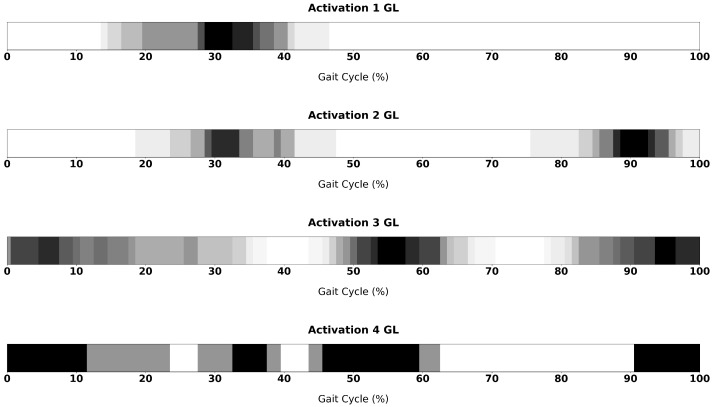
Temporal distribution of GL activation across the gait cycle for subjects exhibiting one to four activation intervals. Each row represents an activation modality, while the grayscale maps indicate the density of ON periods across the normalised gait cycle (%GCT). Darker regions correspond to higher activation frequency among subjects.

Figure 10 and Figure 11 show grayscale activation maps representing the temporal distribution of muscle activations across all processed steps, for TA and GL muscles respectively. The horizontal axis corresponds to a single gait cycle, normalised from 0% to 100%, while the grayscale intensity encodes the number of trials where the activation was registered: white regions indicate time intervals where no activation was detected in any trial, whereas black regions indicate time intervals where activation was detected in all trials. Intermediate gray levels correspond to proportional frequencies. These images provide an intuitive visual summary of how activations are temporally clustered within the gait cycle and allow the reader to identify recurrent regions of activity as well as variability across cycles.

In Table 1 and Table 2, the activation onset and offset timing of the TA muscle and the GL muscle are shown respectively, regarding the different activation modes.

For the TA, across all identified activation patterns, an activation is consistently observed in the terminal swing phase, marking the end of the gait cycle. In the four-activation pattern, this activation extends across the foot-strike event and continues into the initial stance phase, corresponding to the beginning of the subsequent gait cycle. A second activation is present in all patterns except the single-activation one, occurring shortly after the mid-cycle (approximately between 50–60%) and characterised by a short duration. In the three-activation pattern, an additional burst appears at approximately one-third of the gait cycle, whereas in the four-activation pattern a further activation is detected between 60–70% of the gait cycle.

For the GL, in the single-activation pattern, the activation is localised between 30–40% of the gait cycle. This activation is preserved in the two-activation pattern, where it is accompanied by a second activation occurring near the end of the cycle (around 90%). The most common pattern is the three-activation one, in which activations are distributed at the beginning, middle, and end of the gait cycle. The four-activation pattern can be interpreted as a combination of this distribution with the activation recorded in the first two patterns. The activations observed at the end of the gait cycle and at its beginning correspond to a continuous activation spanning the foot-strike event that separates successive gait cycles.

In Table 3 and Table 4, the duration and area of the TA and GL, respectively, are presented for each of the registered activation modes. These tables highlight in detail the time duration of specific activations in percentage of the gait cycle time, and the area under the signal during activation in percentage of the entire area of signal during the gait cycle.

## 4. Discussion

The wearable system presented in this study proved to be an effective tool for assessing muscle activation patterns during gait. As reported, all participants successfully completed every walking trial without discomfort or alterations of natural gait. These outcomes are consistent with our previous findings about comfort of the textile sensorised band [10] and studies on textile electrodes and wearable e-textile systems, which highlight the comfort, stability, and suitability of soft fabric-based sensors for ambulatory EMG monitoring [19]. Given its lightweight design, wireless communication and textile integration, the system demonstrates potential for clinical use as well as remote monitoring and telerehabilitation.

Despite its advantages, the system requires a certain level of assistance for correct placement. Proper alignment of the textile electrodes and secure attachment of the acquisition unit demanded both time and the involvement of an operator. Similar challenges have been reported in the deployment of wearable EMG systems, where correct electrode positioning remains a key requirement for signal reliability [20].

As expected, the TA displayed consistent activation in terminal swing across all identified patterns, a well-established feature related to toe clearance and controlled foot placement, often accompanied by brief secondary bursts in multi-activation modes at around 50–60% of the gait cycle. In the four-activation pattern the principal activation extends across foot strike into early stance, which agrees with reports describing primary TA activity at late swing/initial contact and early stance [21,22,23]. In a limited number of trials (5%) an additional bursts appeared in the late stance phase. These findings could be justified by recent literature suggesting that TA recruitment is modulated by subtle biomechanical demands and may include multiple short bursts contributing to ankle stabilization [21,24,25].

The TA area values, generally modest in magnitude, are consistent with phasic dorsiflexor involvement. The variability in onset, offset and activation area observed across subjects and trials reflects individual movement strategies, which can be influenced by gait speed, neuromuscular control, and posture.

For the GL, a principal activation region was observed between mid-stance and push-off, consistent with its role in propulsion and ankle stabilization [25,26]. Multi-activation patterns indicated additional bursts near terminal swing and early stance, suggesting preparatory or stabilizing contributions. These observations align with consolidated descriptions of distal-leg muscle timing during walking and with reports that sensitive detection methods can reveal short, context-dependent bursts beyond the canonical pattern [25,27]. Area values for GL activations were small and brief, in agreement with the known phasic nature of the muscle. Minor differences in area between activation modes may reflect variations in push-off mechanics or inter-trial adjustments. Since EMG amplitude is strongly influenced by gait conditions, comparisons must consider relevant contextual factors such as walking speed or environmental constraints [28,29].

A key finding of this work is the substantial inter- and intra-subject variability in both the number of bursts (from one to four per gait cycle) and their onset/offset timing. The literature strongly supports interpreting this variability as physiological rather than artifactual: although TA and GL present robust core timing (TA at early stance and late swing and GL at mid-stance/push-off), the specific pattern types (single or multiple bursts), and the extent of co-contractions vary systematically with age, task constraints, analysis methodology, and individual gait strategies [25,30,31]. Actually faster walking increases TA and GL amplitudes, and treadmill walking with fixed speed can produce greater distal limb activation than overground gait [24,28]. Partial unloading also modulates neuromuscular recruitment, with significant changes occurring especially under altered load-bearing conditions [32,33]. Moreover aging can modulate lower limb muscle activity: older adults exhibit increased ankle coactivation during gait, likely as a compensatory stability mechanism [34]. Also the environment has been proven to influence neuromuscular strategies, walking on different surface types or under challenging environmental conditions modifies the timing and amplitude of muscle bursts [29]. The present findings reflect this multifactorial variability, confirming that healthy gait does not follow a single conventional EMG pattern but a spectrum of plausible activation strategies. Due to this high variability confirmed in studies, Di Nardo and co-authors suggested considering the pattern common for all the detected activation modalities as relevant while considering the other bursts as variations due to control strategies [22]. Furthermore, a recent long-duration, multi-channel dataset on healthy adults (5-min overground walking) demonstrates that natural, long recordings expose genuine within-subject variability across strides and recommends analyzing extended trials to capture adaptive recruitment strategies under small intrinsic or extrinsic perturbations [27]. However, a detailed analysis of these contributing factors was not the primary aim of the present work, and dedicated studies conducted under controlled experimental conditions would be necessary to systematically investigate how such variables influence neuromuscular activation strategies during gait.

## 5. Conclusions

The present work aimed to present and evaluate a multisensor wearable system embedding textile-based sEMG electrodes and pressure sensors for integration in gait analysis, and to assess its feasibility in a cohort of healthy volunteers. Results proved system suitability for acquiring physiological signals during walking, enabling the identification and characterisation of muscle activation patterns of foot dorsiflexion muscles TA and GL. All participants successfully completed the walking tasks without discomfort or alterations in natural gait, thereby confirming that the proposed system is both functional and well tolerated, and that it can potentially support the detailed examination of muscular activation during locomotion.

The activation patterns identified during walking task may represent outcomes for the detection of pathological deviations. Abnormal activations of the TA, for instance reduced or absent terminal-swing bursts, are generally associated with foot drop and peroneal neuropathies. Prolonged or erratic activity of the gastrocnemius is frequently observed in neurological conditions such as stroke, spastic paresis and Parkinson’s disease. Wearable systems such as the one proposed here, which are capable of detecting short activation intervals and variations in timing, have the potential to support early screening, clinical monitoring, and the personalisation of rehabilitation and assistive interventions.

Notwithstanding the foregoing, the present study is subject to several limitations. The sample size was modest and lacked demographic diversity, which constrained the generalizability of the findings. Furthermore, the donning procedure demanded a significant investment of time, necessitated precise alignment of the sensing components, and required the assistance of an operator to ensure reliable electrode–skin contact and system stability. This dependency consequently diminishes the feasibility of achieving complete autonomy in domestic or remote monitoring scenarios. Consequently, future developments should concentrate on simplifying the setup, enhancing the self-alignment of textile electrodes, and automating calibration procedures to improve usability outside controlled environments.

Further research should also target validation in larger and more diverse populations, including individuals with gait impairments. In addition, such research should incorporate comparisons with gold-standard EMG systems. Finally, harmonisation of EMG processing methods, such as normalisation, envelope extraction, and activation-detection thresholding, will be essential to improve reproducibility and facilitate clinical translation of wearable multisensor gait analysis systems.

Another limitation concerns the sensitivity of EMG signal quality to electrode placement. Although the sleeve design and SENIAM-based positioning reduce variability, prolonged use or donning by inexperienced operator may lead to sensor misalignment, affecting data reliability. Future developments should therefore focus on optimizing electrode placement exploring solutions such as self-aligning textile structures, adaptive fastening systems, and/or real-time impedance monitoring to detect and correct non-optimal contact. These improvements will be essential for ensuring consistent signal acquisition in long-term studies and remote monitoring scenarios.

## Figures and Tables

**Table 1:** Activation intervals of the TA muscle related to the number of activations, expressed as mean(SD) and normalised to the percentage of the GCT.

	First Activation		Second Activation		Third Activation		Fourth Activation	
Modality	ON	OFF	ON	OFF	ON	OFF	ON	OFF
1-activation	80.7 (3.9)	93.6 (2.5)						
2-activations	49.5 (6.2)	60.4 (4.8)	84.1 (4.9)	97.5 (3.3)				
3-activations	20.5 (10.6)	35.0 (4.2)	47.0 (2.8)	57.5 (2.1)	73.5 (10.6)	94.0 (7.1)		
4-activations	0.0 (0.0)	26.7 (4.0)	44.6 (3.8)	56.3 (3.1)	61.0 (2.0)	74.0 (1.0)	89.3 (1.2)	100.0 (0.0)

**Table 2:** Activation intervals of the GL muscle related to the number of activations, expressed as mean(SD) and normalised to the percentage of the GCT.

	First Activation		Second Activation		Third Activation		Fourth Activation	
Modality	ON	OFF	ON	OFF	ON	OFF	ON	OFF
1-activation	23.3 (6.7)	37.4 (5.1)						
2-activations	28.1 (6.1)	38.6 (5.0)	85.0 (4.5)	95.3 (2.4)				
3-activations	3.6 (7.9)	21.5 (11.2)	49.5 (3.0)	62.1 (4.7)	86.3 (5.6)	97.9 (2.7)		
4-activations	0.0 (0.0)	17.0 (8.5)	30.5 (3.5)	38.0 (1.4)	45.0 (1.4)	60.5 (2.1)	91.0 (0.0)	100.0 (0.0)

**Table 3:** Time duration and activation area of the TA muscle for the first condition, reported as mean(SD). Time duration is expressed as a percentage of the GCT, while the activation area represents the area under the curve during the activation interval, expressed as a percentage of the total area of the averaged sEMG signal.

	First Activation		Second Activation		Third Activation		Fourth Activation	
Modality	Duration	Area	Duration	Area	Duration	Area	Duration	Area
1-activation	13.3 (3.4)	24.5 (6.4)						
2-activations	10.9 (5.4)	15.4 (9.8)	13.4 (3.2)	20.5 (5.2)				
3-activations	14.5 (14.8)	15.6 (17.6)	10.5 (0.7)	11.1 (0.5)	20.5 (3.5)	29.2 (6.0)		
4-activations	26.7 (4.0)	36.7 (2.9)	11.7 (2.9)	10.8 (2.8)	13.0 (1.7)	13.1 (3.4)	9.7 (1.2)	12.5 (1.9)

**Table 4:** Time duration and activation area of the GL muscle for the first condition, reported as mean(SD). Time duration is expressed as a percentage of the GCT, while the activation area represents the area under the curve during the activation interval, expressed as a percentage of the total area of the averaged sEMG signal.

	First Activation		Second Activation		Third Activation		Fourth Activation	
Modality	Duration	Area	Duration	Area	Duration	Area	Duration	Area
1-activation	14.1 (3.4)	25.5 (4.8)						
2-activations	10.4 (3.8)	16.1 (7.5)	10.3 (3.9)	16.3 (6.7)				
3-activations	17.9 (9.6)	22.0 (10.0)	12.5 (4.7)	14.8 (6.8)	11.7 (5.2)	16.1 (8.1)		
4-activations	17.0 (8.5)	22.0 (6.6)	7.5 (5.0)	6.8 (5.5)	15.5 (3.5)	18.6 (3.4)	8.0 (0.0)	10.4 (0.7)

## Data Availability

Data available on request due to privacy and legal restrictions.

## Abbreviations

The following abbreviations are used in this manuscript:

BLE	Bluetooth Low Energy
CRS	Comfort Rating Scale
ECG	Electrocardiography
EMG	Electromyography
GCT	Gait Cycle Time
GL	Gastrocnemius Lateralis
IMU	Inertial Measurement Unit
LiPo	Lithium Polymer
sEMG	Surface Electromyography
SENIAM	Surface Electromyography for the Non-Invasive Assessment of Muscles
TA	Tibialis Anterior

## References

[1] Chen S., Lach J., Lo B., Yang G.Z. (2016). Toward Pervasive Gait Analysis with Wearable Sensors: A Systematic Review. IEEE J. Biomed. Health Inform..

[2] Wimalaratna H.S.K., Tooley M.A., Churchill E., Preece A.W., Morgan H.M. (2002). Quantitative surface EMG in the diagnosis of neuromuscular disorders. Electromyogr. Clin. Neurophysiol..

[3] Steele K.M., Munger M.E., Peters K.M., Shuman B.R., Schwartz M.H. (2019). Repeatability of electromyography recordings and muscle synergies during gait among children with cerebral palsy. Gait Posture.

[4] Mileti I., Zampogna A., Santuz A., Asci F., Del Prete Z., Arampatzis A., Palermo E., Suppa A. (2020). Muscle Synergies in Parkinson’s Disease. Sensors.

[5] Cifrek M., Medved V., Tonković S., Ostojić S. (2009). Surface EMG based muscle fatigue evaluation in biomechanics. Clin. Biomech..

[6] Tropea P., Monaco V., Coscia M., Posteraro F., Micera S. (2013). Effects of early and intensive neuro-rehabilitative treatment on muscle synergies in acute post-stroke patients: A pilot study. J. NeuroEng. Rehabil..

[7] Smith L.H., Hargrove L.J. Comparison of surface and intramuscular EMG pattern recognition for simultaneous wrist/hand motion classification. Proceedings of the 2013 35th Annual International Conference of the IEEE Engineering in Medicine and Biology Society (EMBC).

[8] Farrell T.R., Weir R.F.F. (2008). A Comparison of the Effects of Electrode Implantation and Targeting on Pattern Classification Accuracy for Prosthesis Control. IEEE Trans. Biomed. Eng..

[9] Al-Ayyad M., Owida H.A., De Fazio R., Al-Naami B., Visconti P. (2023). Electromyography Monitoring Systems in Rehabilitation: A Review of Clinical Applications, Wearable Devices and Signal Acquisition Methodologies. Electronics.

[10] Amitrano F., Coccia A., Pagano G., Biancardi A., Tombolini G., Marsico V., D’Addio G. (2024). Measuring Surface Electromyography with Textile Electrodes in a Smart Leg Sleeve. Sensors.

[11] Winter D., Yack H. (1987). EMG profiles during normal human walking: Stride-to-stride and inter-subject variability. Electroencephalogr. Clin. Neurophysiol..

[12] Stewart J.D. (2008). Foot drop: Where, why and what to do?. Pract. Neurol..

[13] Amitrano F., Donisi L., Coccia A., Biancardi A., Pagano G., D’Addio G. Experimental Development and Validation of an E-Textile Sock Prototype. Proceedings of the 2020 IEEE International Symposium on Medical Measurements and Applications (MeMeA).

[14] Amitrano F., Coccia A., Donisi L., Pagano G., Cesarelli G., D’Addio G. Gait Analysis using Wearable E-Textile Sock: An Experimental Study of Test-Retest Reliability. Proceedings of the 2021 IEEE International Symposium on Medical Measurements and Applications (MeMeA).

[15] Cesarelli M., Bifulco P., Bracale M. (2000). Study of the control strategy of the quadriceps muscles in anterior knee pain. IEEE Trans. Rehabil. Eng..

[16] Carvalho C.R., Fernández J.M., del Ama A.J., Oliveira Barroso F., Moreno J.C. (2023). Review of electromyography onset detection methods for real-time control of robotic exoskeletons. J. NeuroEng. Rehabil..

[17] Micera S., Vannozzi G., Sabatini A., Dario P. (2001). Improving detection of muscle activation intervals. IEEE Eng. Med. Biol. Mag..

[18] Drapała J., Brzostowski K., Szpala A., Rutkowska-Kucharska A. (2012). Two stage EMG onset detection method. Arch. Control Sci..

[19] Vidhya C.M., Maithani Y., Singh J.P. (2023). Recent Advances and Challenges in Textile Electrodes for Wearable Biopotential Signal Monitoring: A Comprehensive Review. Biosensors.

[20] Dotti G., Ghislieri M., Castagneri C., Agostini V., Knaflitz M., Balestra G., Rosati S. (2024). An open-source toolbox for enhancing the assessment of muscle activation patterns during cyclical movements. Physiol. Meas..

[21] Kwak S.T., Chang Y.H. (2023). Fascicle dynamics of the tibialis anterior muscle reflect whole-body walking economy. Sci. Rep..

[22] Di Nardo F., Ghetti G., Fioretti S. (2013). Assessment of the activation modalities of gastrocnemius lateralis and tibialis anterior during gait: A statistical analysis. J. Electromyogr. Kinesiol..

[23] Di Nardo F., Mengarelli A., Ghetti G., Fioretti S., Roa Romero L.M. (2014). Statistical Analysis of EMG Signal Acquired from Tibialis Anterior during Gait. XIII Mediterranean Conference on Medical and Biological Engineering and Computing 2013.

[24] Miura K., Kadone H., Koda M., Nakayama K., Kumagai H., Nagashima K., Mataki K., Fujii K., Noguchi H., Funayama T. (2018). Visualization of walking speed variation-induced synchronized dynamic changes in lower limb joint angles and activity of trunk and lower limb muscles with a newly developed gait analysis system. J. Orthop. Surg..

[25] Li W., Li Z., Qie S., Yang H., Chen X., Liu Y., Li Z., Zhang K. (2020). Analysis of the activation modalities of the lower limb muscles during walking. Technol. Health Care.

[26] Flux E., Mooijekind B., Bar-On L., Van Asseldonk E.H., Buizer A.I., Van Der Krogt M.M. (2024). Relation between stretch and activation of the medial gastrocnemius muscle during gait in children with cerebral palsy compared to typically developing children. J. Electromyogr. Kinesiol..

[27] Di Nardo F., Morbidoni C., Iadarola G., Spinsante S., Fioretti S. (2025). Long duration multi-channel surface electromyographic signals during walking at natural pace: Data acquisition and analysis. PLoS ONE.

[28] Nymark J.R., Balmer S.J., Melis E.H., Lemaire E.D., Millar S. (2005). Electromyographic and kinematic nondisabled gait differences at extremely slow overground and treadmill walking speeds. J. Rehabil. Res. Dev..

[29] Lebleu J., Parry R., Bertouille C., De Schaetzen M., Mahaudens P., Wallard L., Detrembleur C. (2021). Variations in Patterns of Muscle Activity Observed in Participants Walking in Everyday Environments: Effect of Different Surfaces. Physiother. Can..

[30] Agostini V., Nascimbeni A., Gaffuri A., Imazio P., Benedetti M., Knaflitz M. (2010). Normative EMG activation patterns of school-age children during gait. Gait Posture.

[31] Kameyama O., Ogawa R., Okamoto T., Kumamoto M. (1990). Electric discharge patterns of ankle muscles during the normal gait cycle. Arch. Phys. Med. Rehabil..

[32] Kolářová B., Krobot A., Polehlová K., Hluštík P., Richards J.D. (2016). Effect of Gait Imagery Tasks on Lower Limb Muscle Activity with Respect to Body Posture. Percept. Mot. Skills.

[33] Haddas R., Belanger T. (2017). Clinical Gait Analysis on a Patient Undergoing Surgical Correction of Kyphosis from Severe Ankylosing Spondylitis. Int. J. Spine Surg..

[34] Schmitz A., Silder A., Heiderscheit B., Mahoney J., Thelen D.G. (2009). Differences in lower-extremity muscular activation during walking between healthy older and young adults. J. Electromyogr. Kinesiol..

